# Predicting treatment response in multicenter non-small cell lung cancer patients based on federated learning

**DOI:** 10.1186/s12885-024-12456-7

**Published:** 2024-06-05

**Authors:** Yuan Liu, Jinzao Huang, Jyh-Cheng Chen, Wei Chen, Yuteng Pan, Jianfeng Qiu

**Affiliations:** 1 School of Radiology, Shandong First Medical University and Shandong Academy of Medical Sciences, Taian, China; 2 School of Radiology, Second Affiliated Hospital of Shandong First Medical University and Shandong Academy of Medical Sciences, Taian, China; 3 Department of Radiology, Cathay General Hospital, Taipei, China; 4 Department of Biomedical Imaging and Radiological Sciences, National Yang-Ming Chiao- Tung University, Taipei, China; 5 Department of Biomedical Imaging and Radiological Science, China Medical University, Taichung, China

**Keywords:** Non-small cell lung cancer, Chemotherapy and radiotherapy, Federated learning, Treatment response

## Abstract

**Background:**

Multicenter non-small cell lung cancer (NSCLC) patient data is information-rich. However, its direct integration becomes exceptionally challenging due to constraints involving different healthcare organizations and regulations. Traditional centralized machine learning methods require centralizing these sensitive medical data for training, posing risks of patient privacy leakage and data security issues. In this context, federated learning (FL) has attracted much attention as a distributed machine learning framework. It effectively addresses this contradiction by preserving data locally, conducting local model training, and aggregating model parameters. This approach enables the utilization of multicenter data with maximum benefit while ensuring privacy safeguards. Based on pre-radiotherapy planning target volume images of NSCLC patients, a multicenter treatment response prediction model is designed by FL for predicting the probability of remission of NSCLC patients. This approach ensures medical data privacy, high prediction accuracy and computing efficiency, offering valuable insights for clinical decision-making.

**Methods:**

We retrospectively collected CT images from 245 NSCLC patients undergoing chemotherapy and radiotherapy (CRT) in four Chinese hospitals. In a simulation environment, we compared the performance of the centralized deep learning (DL) model with that of the FL model using data from two sites. Additionally, due to the unavailability of data from one hospital, we established a real-world FL model using data from three sites. Assessments were conducted using measures such as accuracy, receiver operating characteristic curve, and confusion matrices.

**Results:**

The model’s prediction performance obtained using FL methods outperforms that of traditional centralized learning methods. In the comparative experiment, the DL model achieves an AUC of 0.718/0.695, while the FL model demonstrates an AUC of 0.725/0.689, with real-world FL model achieving an AUC of 0.698/0.672.

**Conclusions:**

We demonstrate that the performance of a FL predictive model, developed by combining convolutional neural networks (CNNs) with data from multiple medical centers, is comparable to that of a traditional DL model obtained through centralized training. It can efficiently predict CRT treatment response in NSCLC patients while preserving privacy.

**Supplementary Information:**

The online version contains supplementary material available at 10.1186/s12885-024-12456-7.

## Introduction

Non-Small Cell Lung Cancer (NSCLC), as one of the most common cancers, exhibits high incidence and mortality rates [1]. For patients ineligible for radical surgery, the combination of radiotherapy and chemotherapy represents their primary treatment option [2]. The assessment of treatment response, which relates to the quality of survival and the effectiveness of treatment, is the key to enhancing the prognosis of patients [3]. Within the same disease stage, patients exhibit varying responses to radiotherapy. Some experience tumor shrinkage, while others manifest signs of tumor progression [4]. Current criteria for Response Evaluation Criteria in Solid Tumors (RECIST) [5] judge treatment effectiveness based on tumor size, shape, and growth rate. However, these criteria usually take time to produce observable changes, making it challenging to provide real-time therapeutic feedback during the early stages [6]. Therefore, an urgent demand exists for a method capable of delivering real-time efficacy predictions to facilitate more informed treatment decisions.

With the advancement of artificial intelligence, the prediction of treatment response has become efficient and accurate within the precision medicine. Liu et al. [7] used MRI images to predict treatment response to chemotherapy in patients with nasopharyngeal carcinoma. Sammut et al. [8] constructed a model based on clinical, digital pathology, genomic and transcriptomic profiles to predict pathological complete responses in breast cancer cases. Xu et al. [9] constructed a deep learning (DL) model by CT images of NSCLC patients to predicted survival and cancer specific outcomes. However, highly accurate models require a wide variety of datasets. In practice, the medical image data held by each institution tends to be limited and fragmented, and the increasing awareness of data privacy makes multi-center data collection difficult. However, there are three main problems. To begin, training high-precision models often requires pooling data together in most studies [10]. Yet, medical image data held by each institution tends to be limited and fragmented, making data collection challenging. Additionally, the increasing awareness of data privacy makes multi-center data collection difficult [11]. Moreover, data centralization may bring the risk of data leakage.

Meanwhile, Federated Learning (FL) [12, 13] has garnered substantial interest within the medical domain as an emerging machine learning technique [14]. In multicenter medical research, data is typically siloed across various medical institutions and research centers, and sharing data across institutions becomes infeasible due to privacy and regulatory constraints. In contrast, FL presents a novel avenue. It allows multiple data holders to collaboratively construct machine learning models without sharing raw data [15, 16]. This approach protects patient privacy by performing model training on local devices and sharing only model parameters. Pati et al. [17] employed FL to detect sub-compartment boundaries of glioblastoma. Similarly, Islam et al. [18] leveraged FL to build CNN architectures to identify brain tumors in MRI images. Likewise, Yan et al. [19] used FL for the automatic detection of COVID-19 lesions in images. However, existing research in this area has significant limitations, particularly regarding diversity. The evaluation of efficacy using FL has not been fully explored. Furthermore, there are limitations in applying FL in real-world environments. Most studies have primarily focused on experiments in simulation environment, lacking validation in actual medical settings. This limits the practical application value of FL.

The objective of this study aims to address this gap by exploring the application of FL in real-world settings for predicting the response to radiotherapy treatment in a multicenter cohort of NSCLC patients. We’ve set up a privacy-preserving data analysis framework through cross-institutional federated learning in collaboration with multiple medical centers. It allows medical image data from different centers to collaborate in training efficacy prediction models while ensuring data privacy. Through this study, we hope to offer novel insights into the treatment of NSCLC patients, paving the way for innovative prospects in multicenter collaborative research. Ultimately, our goal is to assist physicians in enhancing the precision of treatment response assessment and optimizing cancer treatment outcomes.

## Methods

### Patients

Image data of NSCLC patients with planning target volume images made within 1–3 days before radiotherapy at four hospitals from 2016 to 2022 was reviewed. The research received approval from the Ethics Committee of the Affiliated Hospital of Shandong First Medical University (No. SB-KJCX2101) and the Ethics Committee of the Xiangya Hospital of Central South University (No. 202,207,167). The requirement of written informed consent was waived.

The inclusion criteria were as follows: (I) 18 years of age or older; (II) primary NSCLC; (III) CRT treatment; (IV) A CT scan both prior to and within 5 months after completing the identical course of CRT; (IV) no history of surgical removal of the tumor for treatment.

The exclusion criteria were as follows: (I) patients with incomplete clinical or imaging information; (II) failure to complete the intended treatment planning protocol; (III) patients with no information on efficacy assessment after treatment; and (IV) patients with other primary tumors. After exclusion, the study ultimately included 245 patients who met the specified conditions. Among them, 102 cases originated from an affiliated hospital of Shandong First Medical University (Hospital A), 42 cases originated from Xiangya Hospital of Central South University (Hospital B), 32 cases were from a different affiliated hospital of Shandong First Medical University (Hospital C), and 69 cases were from Cathay General Hospital (Hospital D).

According to RECIST, the outcome of patients’ treatment response was assessed by two proficient radiologists at each center, who analyzed the CT images taken prior to and after CRT administration. Patients with complete response (CR) and partial response (PR) who demonstrated a positive biological effect on treatment were categorized as responsive. Conversely, patients with stable disease (SD) and progressive disease (PD) who demonstrated a limited or negative biological effect were categorized as nonresponsive [7, 20]. The protocols of the scanning in the four hospitals are shown in Supplementary 1.

### Data preprocessing

Observing the collected images of planning target volume, there were differences in the naming of segmentation target volume and endangered organs in different hospitals, we first imported the raw data file and RT struct file into 3D-slicer (4.11) [21] and manually selected the region of interest (ROI) named GTV in the list of names of RT struct file. The raw data and ROI were then converted to nii format images. Due to the differences thickness, the original Dicom and ROI were resampled to 1 × 1 × 1 mm^3^ using B-spline interpolation. The ROI was converted to a 3D binary matrix and the tumor region was calculated. Statistical analysis of tumor sizes at three XYZ levels was conducted, and by referencing previous studies [22–24], a size of 64 × 64 × 64 mm³ was determined (Supplementary 2). Subsequently, 3D patches were cut with the center of the tumor as the origin for input to the model.

To prevent overfitting, increase the data diversity, and bolster the model’s generalization capability, we perform image augmentation on the training data. The methods applied were as follows: [1] Random horizontal flip [2] Random rotation within the − 15° to 15° range [3] Random cropping [4] Brightness, contrast, saturation, and hue set to 0.2.

### Neural network structure

**Fig. 1:**
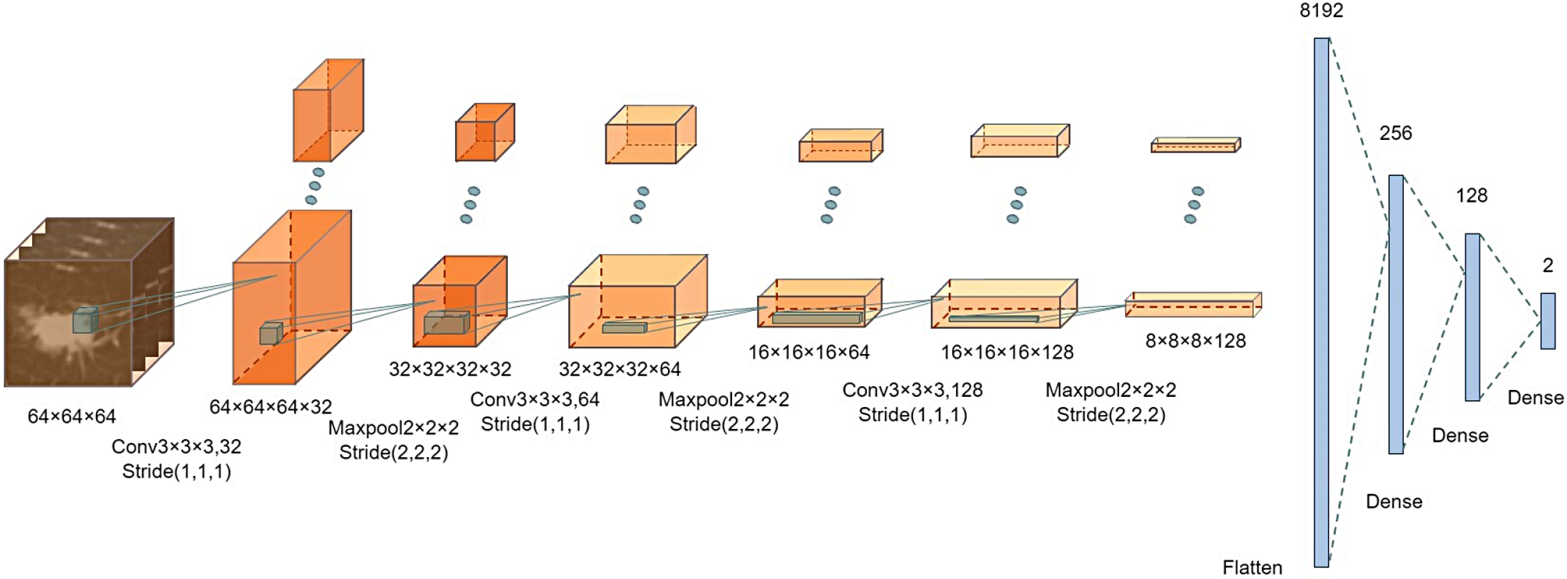
The architecture of conv3DNet. Including convolution layers, max pooling layers, and fully connected layers

A custom 3D convolutional neural network model, conv3DNet, was used in this study. This model was designed from scratch, using a train-from-initialization approach, rather than relying on a pre-trained model structure. The model consists of three convolutional layers, three 3D Max-Pooling pooling layers, two fully-connected layers, and a Softmax layer for final regression to probability. The architecture of the model is shown in Fig. 1.

### Federated learning framework

The FL part of this study was implemented using a FL framework, Flower 1.3.0 [25], which consists of three main modules: server-side, client-side, and strategy [26]. The server-side handles global aggregation, while the client-side manages local training. Within the built-in strategy module, various FL training schemes are embedded, facilitating the selection of the appropriate approach to achieve model parameter aggregation on the server. The framework contains popular FL algorithms such as FedAvg [12] and FedProx [27].

**Fig. 2:**
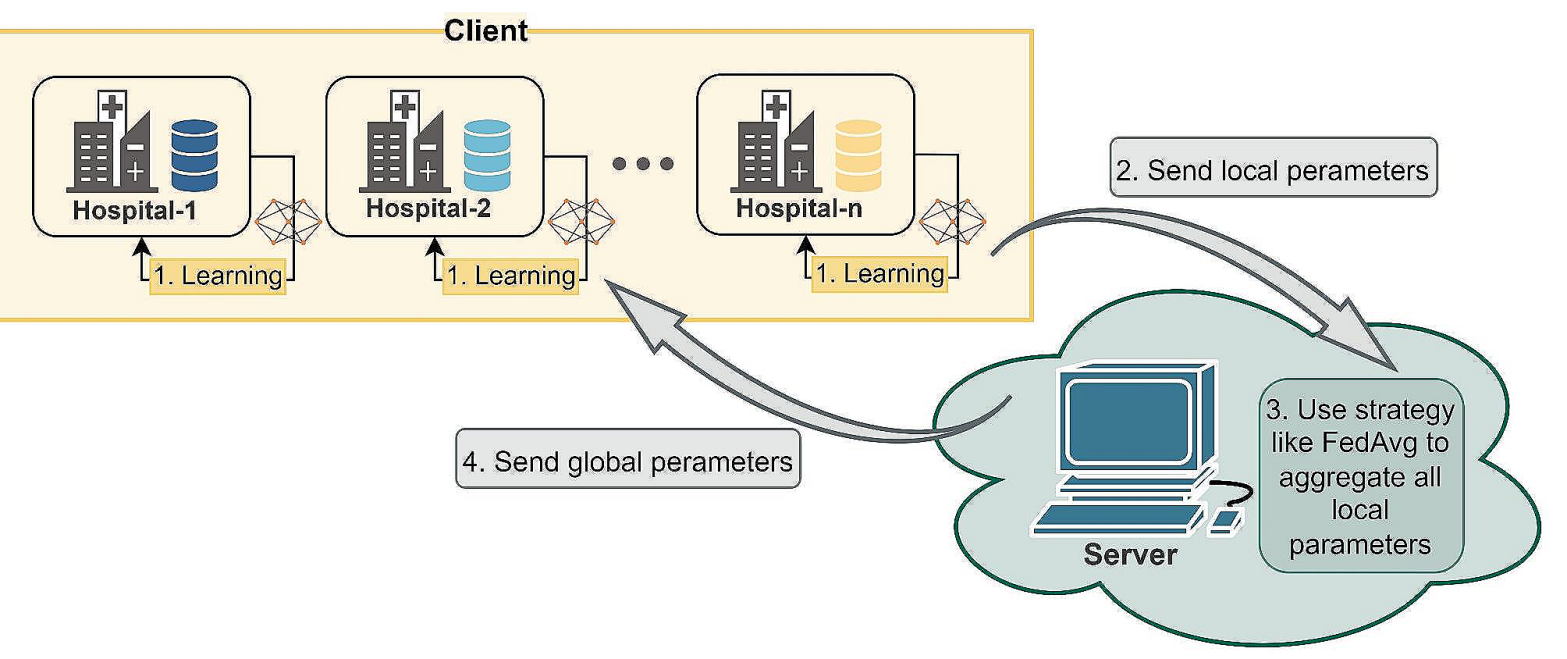
The process of federated learning

The process of federated learning is presented in Fig. 2. First, all clients (participants) wait for the server (central node) to transmit the initial parameters. After receiving the initial parameters, the clients train the model locally using their own training set while the server remains in a waiting state. After each iteration, the client generates a model parameter update. Once a client finishes training, it transmits the model parameters to the server over the network. After receiving the parameters from all clients, the server uses the FedAvg algorithm to aggregate the parameter updates by taking their weighted average. The server then sends the aggregated parameters back to the clients over the network, and the clients train their local model again based on these aggregated parameters. The whole process is iterated repeatedly, with each iteration training the local model on the client, generating parameter updates, transmitting them to the server for aggregation, and obtaining the final global model [15, 28].

### Model construction

In this study, we first built a DL model and a two-client FL model in a simulated environment, comparing their performance to elucidate the advantages of FL. Due to the unavailability of data from hospital D, we conducted a real-world three-client FL model to explore the potential of FL in healthcare applications. The model frameworks all come from conv3Dnet mentioned in 2.3.

#### Federated learning in a simulation environment

Initially, we developed a centralized DL model by dividing the data from hospitals A and B into training and validation sets in a ratio of 7:3. In addition, data from hospital C was used as an external validation set for testing the model, and was not involved in model training or debugging. The training hyperparameters included the following: (I) Batch size set at 8; (II) Learning rate of 0.001; (III) Utilizing the Adam optimizer; and (IV) Training for 100 epochs. The loss function employed was cross entropy.

The data were then utilized to build a FL model. Two clients (Hospital A and B) divided the local data into a training set and a validation set in a 7:3 ratio. The data from Hospital C was used as an external validation set for model testing and was not involved in model training or debugging. We configured the initial global model for both clients and used the Flower framework to communicate with the central server via gRPC to build the FL model. The training hyperparameters included the following: (I) Batch size set at 16; (II) Learning rate of 0.001; (III) Utilizing the SGD optimizer (To assess the initial performance of the FL model, we employ an SGD optimizer capable of fine-tuning the model.); (IV) Conducting 10 communication rounds; and (V) Training for 50 local epoch per client. (Local epoch means that each client trains with its local data before sending model parameters to center server.) The loss function used remained cross entropy.

#### Federated Learning in Real-world environments

**Fig. 3:**
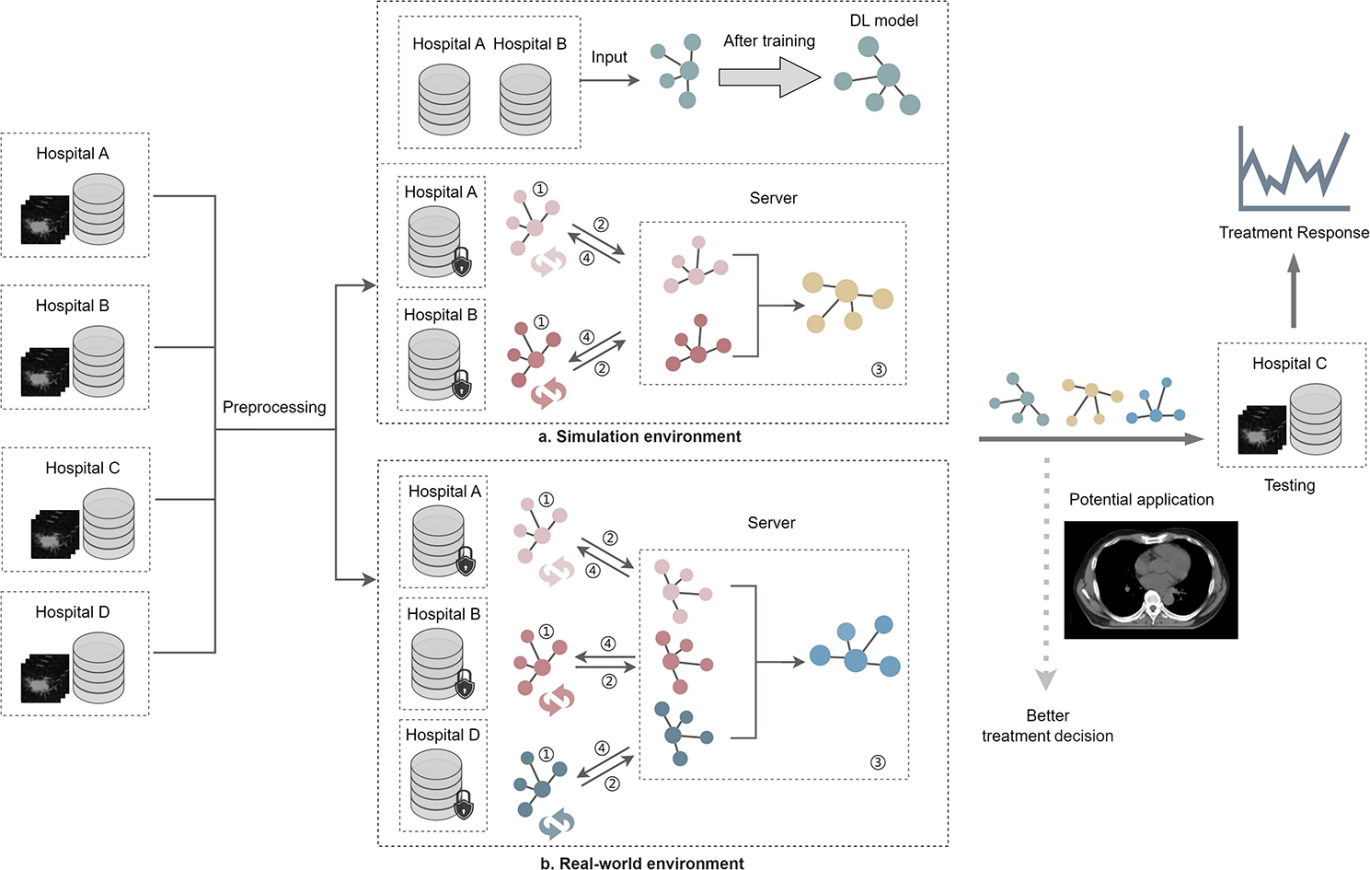
Model training architecture for NSCLC treatment response from CT images. (FL training process: ①Model is trained using local data. ②Model parameters are sent to the server. ③Server aggregation parameters. ④Update parameters.)

The experiment in FL in the real world was continued using the Flower framework. The configuration of the Flower framework to process data from different healthcare organizations and protect data privacy is described. The approach is as follows: Three clients (hospital A, hospital B and hospital D) divided the local data into a training set and a validation set in a 7:3 ratio. The data from hospital C was used as an external validation set for model testing and was not involved in model training and debugging. The model training is all performed on the client side, and the flow is shown in Fig. 3. The training hyper-parameters were as follows: (I) Batch size set at 16 (II) Learning rate of 0.001; (III) Utilizing the Adam optimizer (In real-world environment with higher data distribution and complexity, we use the Adam optimizer, which adaptively adjusts the learning rate for faster and more stable convergence.); (IV) Conducting 10 communication rounds; and (V) Training for 50 local epoch per client. The loss function employed remained cross entropy.

In deep learning, the model’s performance is influenced by the distribution of the dataset. To assess the robustness of the proposed model, a switch to a different dataset for external validation was made, following the method described earlier. In the simulation environment, we used data from hospitals A and C to train the centralized learning model DL2, while two clients (Hospital A and C) were used to train the distributed learning model FL3. In the real world, three clients (Hospital A, C, and D) were used to train the distributed learning model FL4. Experimental parameters are shown in Supplementary 3. Data from Hospital B was used as an external validation set to test models DL2, FL3, and FL4, and was not involved in model training or debugging.

### Experimental environment

Statistical analyses were performed using IBM SPSS Statistics 21.0 software. CPU processor is Intel(R) Core(TM) i5-11500 @2.70 GHz 2.71 GHz, Intel(R) Xeon(R) Silver 4114 CPU @ 2.20 GHz 2.19 GHz. The GPU processor is NVIDIA Quadro P4000; the operating system is 64 bit Windows 10 Professional with python 3.7; and the network model is implemented using a deep learning framework based on Pytorch(1.13.1).

## Results

To train a multicenter well-performing predictive model, a highly diverse dataset is essential. In light of this, we collected data from a total of 245 NSCLC patients from four centers. Among these, 110 cases were classified within the responsive group, while the remaining 135 cases fell into the non-responsive group. A detailed summary of the cohort’s demographic information is presented in Table 1. To ensure no significant differences in patient characteristics across institutions, we conducted statistical analyses using one-way ANOVA, Chi-square tests, and Fisher’s exact test. Results indicated that, aside from gender, all other characteristics were not significantly different across institutions (*p* > 0.05). Although gender differences among the four cohorts were significant (*p* < 0.05), we believe gender does not impact our results as our analysis primarily relies on image features.

**Table 1:** Patient characteristics

Subjects	Hospital A (*n* = 102)	Hospital B (*n* = 42)	Hospital C (*n* = 32)	Hospital D (*n* = 69)	*P* value
R	51	31	17	11	
nR	51	11	15	58	
Gender					*P* < 0.05
Male	82	34	23	36	
Female	20	8	9	33	
Age	64.32 ± 7.81	64.21 ± 10.68	63.96 ± 8.57	68.7 ± 11.4	0.057
Range	47–81	44–83	39–79	37–91	
Histological type					0.269
LUAD	48	17	14	40	
LUSC	54	25	18	29	
Tumor stage					0.120
T1	10	11	3	8	
T2	32	16	10	21	
T3	34	7	11	16	
T4	26	8	8	24	
Node stage					0.114
N0	16	10	7	20	
N1	9	5	6	7	
N2	51	13	13	19	
N3	26	14	6	23	
Metastasis					0.065
M0	66	24	22	32	
M1	36	18	10	37	
Clinical stage					0.187
IIIA	26	7	12	12	
IIIB	36	14	14	26	
IIIC	7	3	2	4	
IV	33	18	4	27	

R, responsive; nR, non-responsive. LUAD, Lung adenocarcinoma; LUSC, Lung squamous cell carcinoma

The performance of centralized DL models (DL1, DL2) and FL models (FL1, FL3) in simulated environments, as well as FL models (FL2, FL4) in the real world, are evaluated using accuracy, specificity, AUC value, and confusion matrix. For a fair comparison, the models are all evaluated using the same test set (Hospital B or C).

**Fig. 4:**
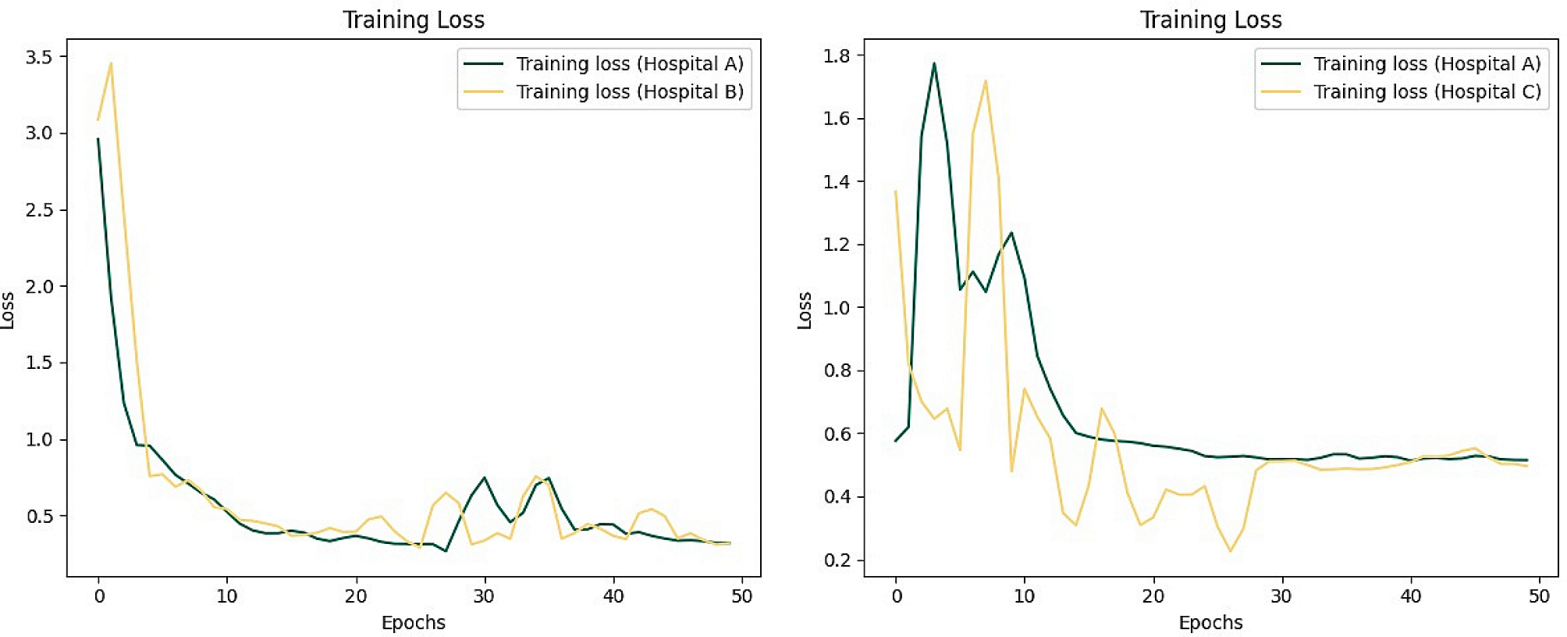
The training loss of the FL model across the two hospitals

**Fig. 5:**
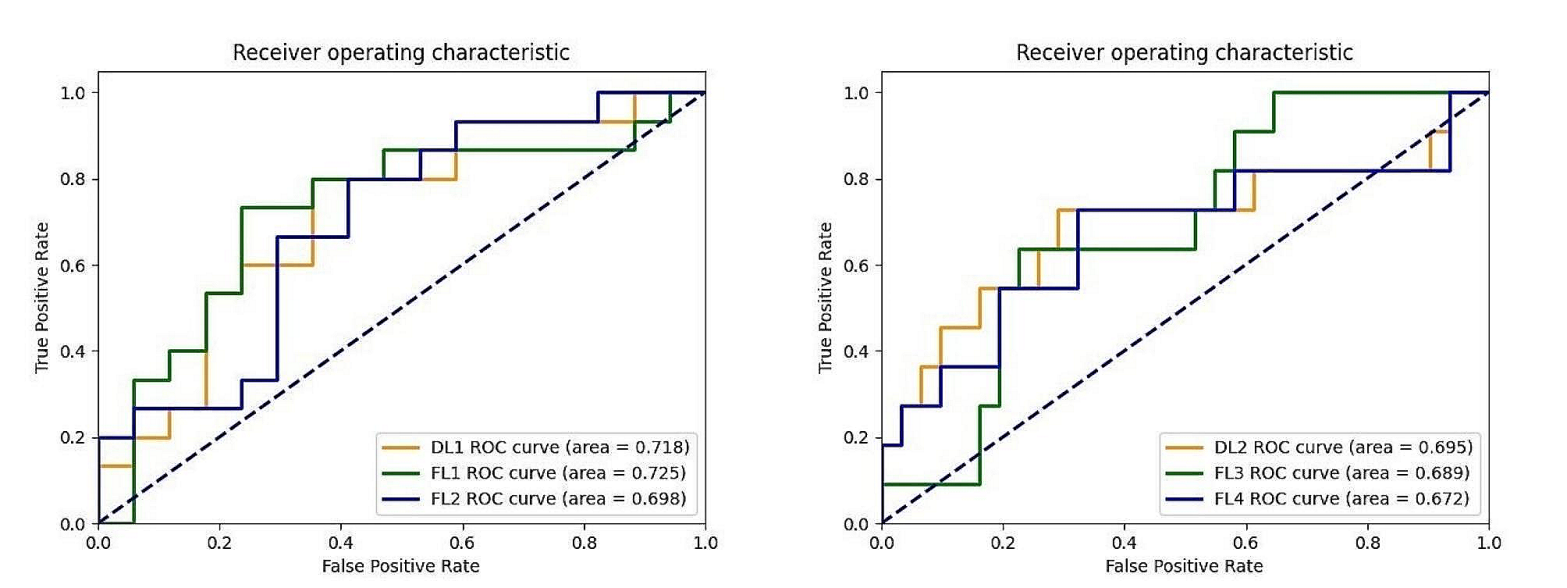
Performance comparison between local and collaborative FL training based on imaging data to predict treatment response in NSCLC patients. ROC curve of three models

**Fig. 6:**
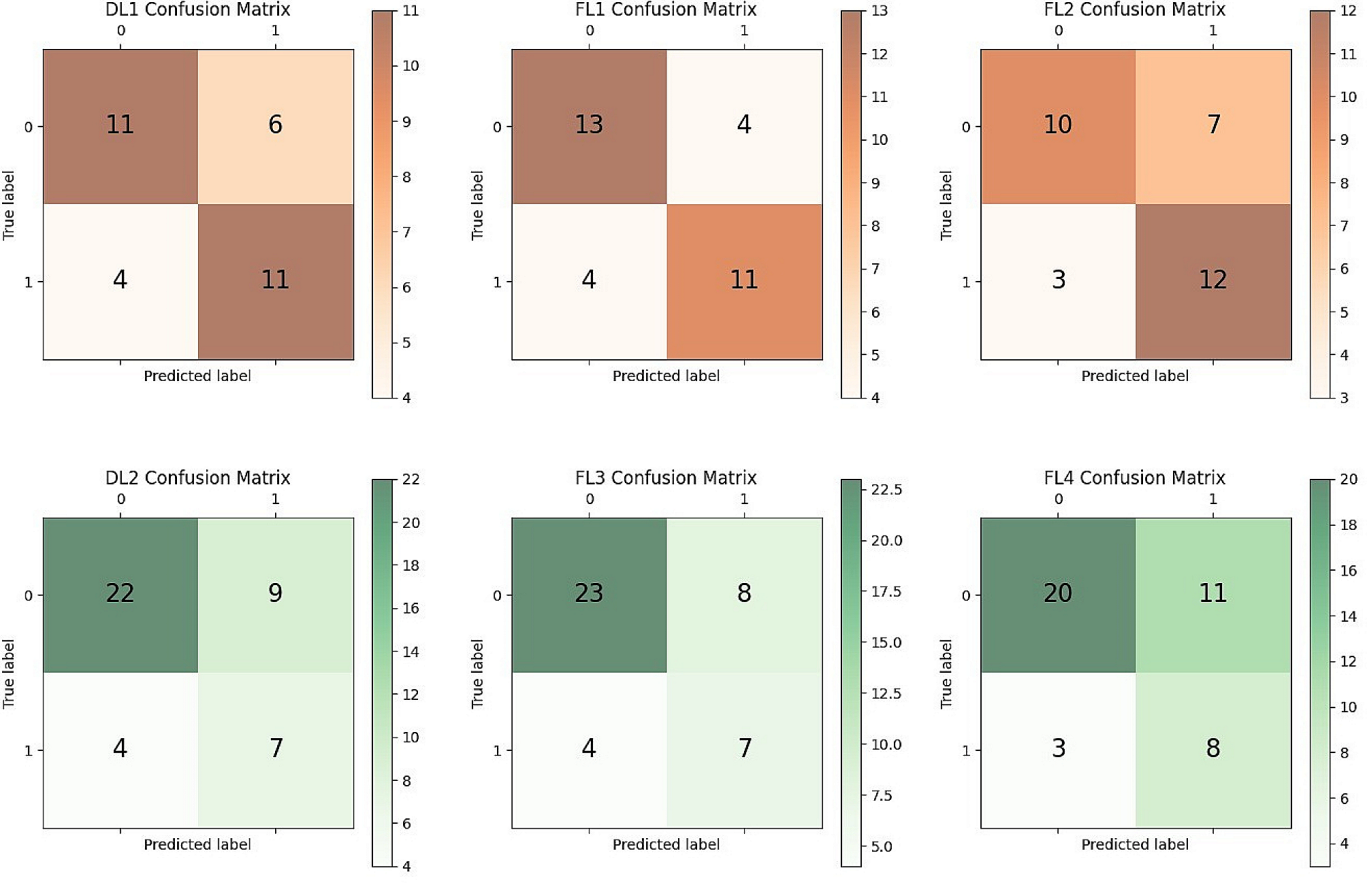
Performance comparison between local and collaborative FL training based on imaging data to predict treatment response in NSCLC patients. Confusion matrix of three models

When validated using the dataset from hospital C in the simulated environment, the centralized DL1 model exhibits an AUC value of 0.718(95% CI: 0.52–0.88). The FL1 model achieves higher AUC value of 0.725(95% CI: 0.55–0.90). In the real-world setting, the FL2 model shows an AUC value of 0.698(95% CI: 0.49–0.87). After switching dataset B for external validation, the DL2 model built in the simulated environment achieved an AUC value of 0.695(95% CI: 0.45–0.90), while the FL3 model achieved an AUC value of 0.689(95% CI: 0.51–0.85). Additionally, the FL4 model built in the real world attained an AUC of 0.672(95% CI: 0.45–0.89). Figure 4 summarizes the training loss of the FL model across the two hospitals. Figures 5 and 6 summarizes the ROC curves and confusion matrices obtained from the above three models. Tables 2 and 3 summarize the performance metrics for our model’s training, validation, and testing.

**Table 2:** Three model’s performance on treatment response prediction using hospital C test set

	Training					Validation					Testing			
	Acc	AUC	Spe	Recall		Acc	AUC	Spe	Recall		Acc	AUC	Spe	Recall
DL1	0.760	0.807	0.789	0.721		0.682	0.720	0.720	0.632		0.688	0.718	0.647	0.733
FL1	0.796	0.761	0.823	0.755		0.718	0.740	0.709	0.674		0.750	0.725	0.765	0.733
FL2	0.734	0.792	0.663	0.762		0.667	0.693	0.669	0.683		0.688	0.698	0.588	0.800

Acc: Accuracy Spe: Specificity. DL1: Centralized deep learning model trained with AB hospital data in simulation environment. FL1: Distributed federated learning model trained with AB hospital data in simulation environment. FL2: Distributed federated learning model trained with ABD hospital data in real-world environment.

**Table 3:** Three model’s performance on treatment response prediction using hospital B test set

	Training					Validation					Testing			
	Acc	AUC	Spe	Recall		Acc	AUC	Spe	Recall		Acc	AUC	Spe	Recall
DL2	0.741	0.771	0.723	0.761		0.732	0.724	0.714	0.750		0.691	0.695	0.710	0.636
FL3	0.736	0.777	0.740	0.733		0.693	0.680	0.683	0.712		0.714	0.689	0.742	0.636
FL4	0.720	0.749	0.657	0.763		0.666	0.705	0.641	0.736		0.667	0.672	0.645	0.727

DL2: Centralized deep learning model trained with AC hospital data in simulation environment. FL3: Distributed federated learning model trained with AC hospital data in simulation environment. FL4: Distributed federated learning model trained with ACD hospital data in real-world environment

## Discussion

In previous multicenter studies, machine learning or deep learning methods are usually employed to construct models using diverse medical imaging data such as CT and MRI. For instance, Cui et al. [29] developed a DL model for predicting individual patient responses to neoadjuvant chemotherapy based on CT images of patients with locally progressed gastric cancer from four hospitals in China. Braman et al. [30] built a machine learning model to predict the ability of neoadjuvant chemotherapy to provide a complete remission of the pathology through MRI images of breast cancer patients. However, these conventional methods typically necessitate data centralization for training, raising concerns about data privacy.

Therefore, methods such as swarm learning (SL) [31] and FL allow for training models in multi-center collaborations without sharing sensitive data. SL is a decentralized machine learning approach that does not require server-coordinated parameters, enabling direct communication between parties through a blockchain network. For instance, Saldanha et al. [32] used SL in a multicenter study to predict gene mutation status and microsatellite instability. Another study employed SL to predict molecular biomarkers for gastric cancer [33], both of which achieved remarkable results.

Unlike SL, FL utilizes a central server for coordination, through which all participants communicate. This method effectively integrates multicenter data while protecting data privacy. For example, Sheller et al. [34] divided the BraTs dataset into 10 simulated institutions to study simulated FL, which aimed to distinguish healthy brain tissue from cancerous tissue. Sadilek et al. [35] conducted several studies on FL in different scenarios to explore its performance. However, many of the FL studies in existing research have been conducted in simulated environments. These studies primarily focus on technological innovations in data security and privacy protection, but lack validation in real healthcare environments. In contrast, our study focuses more on predicting efficacy in practical clinical applications and validates the feasibility of FL in real clinical settings.

In this study, we introduced the Flower FL framework to establish a collaborative multicenter learning model based on 3D CT images for predicting the treatment response of radiotherapy in NSCLC patients. Our research involved a cohort of 245 patients from four different hospitals. We began with a theoretical performance comparison conducted in a simulation environment using data from three of these hospitals. By comparing the performance of a DL model built by a centralized approach with a FL model, our findings support the effectiveness of the FL approach [DL model accuracy = 0.688/0.691, AUC = 0.718/0.695; FL model accuracy = 0.750/0.714, AUC = 0.725/0.689]. In real-world scenarios, in order to more closely match the actual medical application scenarios and to address the challenges of data acquisition and privacy protection, we utilize data from all four hospitals to develop a FL model [FL model accuracy = 0.688/0.667; area under the curve (AUC) = 0.698/0.672].

The left panel of Fig. 4 shows that after 20 epochs, the loss values fluctuate. And the right panel shows that as training proceeds, the losses for both hospitals gradually decrease and stabilize. The relatively smoother loss curve for Hospital A may indicate a larger amount of data at this site, facilitating smoother learning for the model [36]. As demonstrated in Tables 2 and 3; Figs. 5 and 6, since the model weights received by the federated global model are the weighted average of the local model weights from other clients, which are aggregated by the global model and then returned to the client’s local model, each client can benefit from the experiences of the other clients, thus the FL1, FL3 model’s performance, surpasses that of the DL1, DL2 model. The federated learning models FL2 and FL4, trained using data from three clients, exhibited lower metrics compared to FL1 and FL3, which were trained using data from two clients. This discrepancy may attribute to uneven data distribution in hospital D compared to the other two clients. In cases where data distribution differs noticeably among clients, there may be inconsistencies in data distribution during model aggregation. Using fewer clients reduces the likelihood of this data distribution inconsistency. In addition, the performance of the model trained using AC hospital data (with hospital B as the test set) is comparatively lower than that trained using AB hospital data (with hospital C as the test set). In FL, hospital A, which possesses a larger dataset, is assigned a higher weight. Hence, this discrepancy in performance could potentially be attributed to the fact that the data distribution of Hospital C aligns more closely with that of Hospital A compared to Hospital B.

Certain limitations remain in this study. First, the dataset of NSCLC patients used in this paper was relatively small, and the inclusion of data from more medical institutions could potentially enhance the model’s performance. In addition, this study was limited by the sample size, and CR and PR were categorized as treatment with remission, and SD and PD were categorized as treatment without remission for dichotomous studies. In the future, it is hoped that the RECIST criteria will be used to classify efficacy into four classes for more accurate prediction, while expanding and balancing the sample size. Furthermore, this study focused on constructing a CT-based unimodal model using FL, omitting the integration of additional data such as clinical features and pathology image features, which have the potential to enhance the model’s predictive capacity in the context of cancer treatment response. Future studies will aim to expand the size of the dataset, invite more medical institutions to participate, and integrate data from various sources to build a more comprehensive model for the precise prediction of radiotherapy treatment response in NSCLC patients.

## Conclusions

To emphasize the efficacy of a distributed learning approach in a data-private setting, we conducted a study on FL for predicting treatment responses among patients with NSCLC.

We compared traditional DL and FL approaches. Our results show that FL can achieve comparable performance to centralized DL without sharing sensitive data. In addition, we validate the feasibility of FL in real-world applications. We believe that this approach is not only applicable to NSCLC efficacy prediction, but can also be extended to other DL applications for medical image analysis. This research provides an effective approach to address data privacy and collaboration issues in multicenter medical image analysis, which is expected to have a broader impact in clinical applications.

### Electronic supplementary material

Below is the link to the electronic supplementary material.

Supplementary Material 1

Supplementary Material 2

Supplementary Material 3

## Data Availability

The data that support the findings of this study are not openly available due to reasons of sensitivity and are available from the corresponding author upon reasonable request.

## Abbreviations

NSCLC: Non-small cell lung cancer
FL: Federated learning
SL: Swarm learning
CRT: Chemotherapy and radiotherapy
DL: Deep learning
CNNs: Convolutional neural networks
RECIST: Response Evaluation Criteria in Solid Tumors
CR: Complete response
PR: Partial response
SD: Stable disease
PD: Progressive disease
