# Supplementary material for: Predicting treatment response in multicenter non-small cell lung cancer patients based on federated learning
Source: BMC Cancer. 2024 Jun 5;24:688. doi: 10.1186/s12885-024-12456-7 (PMC11155008; doi:10.1186/s12885-024-12456-7)
Supplement: Supplementary file 1 — Supplementary Material 1 [file 12885_2024_12456_MOESM1_ESM.docx]

Supplementary 2 The tumor sizes from the four hospitals at the XYZ level.

| Subjects | Hospital A (n=102) | | | Hospital B (n=42) | | | Hospital C (n=32) | | | Hospital D (n=69) | | |
| --- | --- | --- | --- | --- | --- | --- | --- | --- | --- | --- | --- | --- |
| Axis | X | Y | Z | X | Y | Z | X | Y | Z | X | Y | Z |
| Average | 60.71 | 59.54 | 62.33 | 51.48 | 50.64 | 59.52 | 79.69 | 73.22 | 86.97 | 46.18 | 51.08 | 53.20 |
| Median | 59.00 | 58.00 | 59.00 | 47.00 | 51.00 | 53.00 | 87.50 | 73.50 | 81.50 | 48.00 | 44.00 | 47.00 |
