# Supplementary material for: Predicting treatment response in multicenter non-small cell lung cancer patients based on federated learning
Source: BMC Cancer. 2024 Jun 5;24:688. doi: 10.1186/s12885-024-12456-7 (PMC11155008; doi:10.1186/s12885-024-12456-7)
Supplement: Supplementary file 2 — Supplementary Material 2 [file 12885_2024_12456_MOESM2_ESM.docx]

Supplementary 1 The scanning equipment parameters

| Hospital | Machine brand | Scanning voltage(kVp) | Tube current(mA) | Exposure time(s) | Slice thickness  (mm) | Pixel spacing(mm)^2^ |
| --- | --- | --- | --- | --- | --- | --- |
| A | Philips | 120-130 | 53-400 | 1.8-3.6 | 3-5 | 0.7*0.7-1.0*1.0 |
| B | Siemens | 100-140 | 39-473 | 5.0-10.0 | 3-5 | 0.7*0.7-1.0*1.0 |
| C | Philips | 110-140 | 45-425 | 4.0-8.0 | 3-5 | 0.9*0.9-1.0*1.0 |
| D | Canon | 120 | 110/270 | 5.0 | 5 | 0.48*0.48-0.54*0.54 |
|  | Philips | 120 | 122 | 7.4 | 5 | 0.56*0.56 |
