# Supplementary material for: Predicting treatment response in multicenter non-small cell lung cancer patients based on federated learning
Source: BMC Cancer. 2024 Jun 5;24:688. doi: 10.1186/s12885-024-12456-7 (PMC11155008; doi:10.1186/s12885-024-12456-7)
Supplement: Supplementary file 3 — Supplementary Material 3 [file 12885_2024_12456_MOESM3_ESM.docx]

Supplementary 3 Experimental parameters using ACD hospital as the training dataset

| Model | Batch size | Learning rate | Loss function | optimizer | Epoch | Communication round |
| --- | --- | --- | --- | --- | --- | --- |
| DL2 | 8 | 0.001 | cross entropy | Adam | 100 | --- |
| FL3 | 8 | 0.001 | cross entropy | Adam | 50 | 10 |
| FL4 | 8 | 0.001 | cross entropy | Adam | 50 | 10 |
